# Sarcopenia provides extra value outside the PULP score for predicting mortality in older patients with perforated peptic ulcers

**DOI:** 10.1186/s12877-023-03946-7

**Published:** 2023-05-04

**Authors:** Yu-Hao Wang, Yu-San Tee, Yu-Tung Wu, Chi-Tung Cheng, Chih-Yuan Fu, Chien-Hung Liao, Chi-Hsun Hsieh, Stewart C. Wang

**Affiliations:** 1 Department of General Surgery, Chang Gung Memorial Hospital, No.5, Fuxing St., Guishan Dist, Taoyuan City, 333 Taiwan; 2 Division of Trauma and Emergency Surgery, Department of Surgery, Chang Gung Memorial Hospital, No.5, Fuxing St., Guishan Dist, Taoyuan City, 333 Taiwan; 3 Division of Acute Care Surgery, University of Michigan Medical School, 1301 Catherine St, Ann Arbor, MI USA; 4 Morphomic Analysis Group, University of Michigan, 1301 Catherine St, Ann Arbor, MI USA

**Keywords:** Perforated peptic ulcer, Geriatric, Sarcopenia, PULP score

## Abstract

**Background:**

Perforated peptic ulcer (PPU) remains challenging surgically due to its high mortality, especially in older individuals. Computed tomography (CT)-measured skeletal muscle mass is a effective predictor of the surgical outcomes in older patients with abdominal emergencies. The purpose of this study is to assess whether a low CT-measured skeletal muscle mass can provide extra value in predicting PPU mortality.

**Methods:**

This retrospective study enrolled older (aged ≥ 65 years) patients who underwent PPU surgery. Cross-sectional skeletal muscle areas and densities were measured by CT at L3 and patient-height adjusted to obtain the L3 skeletal muscle gauge (SMG). Thirty-day mortality was determined with univariate, multivariate and Kaplan–Meier analysis.

**Results:**

From 2011 to 2016, 141 older patients were included; 54.8% had sarcopenia. They were further categorized into the PULP score ≤ 7 (*n*=64) or PULP score > 7 group (*n*=82). In the former, there was no significant difference in 30-day mortality between sarcopenic (2.9%) and nonsarcopenic patients (0%; *p*=1.000). However, in the PULP score > 7 group, sarcopenic patients had a significantly higher 30-day mortality (25.5% vs. 3.2%, *p*=0.009) and serious complication rate (37.3% vs. 12.9%, *p*=0.017) than nonsarcopenic patients. Multivariate analysis showed that sarcopenia was an independent risk factor for 30-day mortality in patients in the PULP score > 7 group (OR: 11.05, CI: 1.03-118.7).

**Conclusion:**

CT scans can diagnose PPU and provide physiological measurements. Sarcopenia, defined as a low CT-measured SMG, provides extra value in predicting mortality in older PPU patients.

**Supplementary Information:**

The online version contains supplementary material available at 10.1186/s12877-023-03946-7.

## Background

Within the past 50 years, the age when patients are diagnosed with perforated peptic ulcer (PPU) has shifted from 30-40 years to 60 years or higher [1, 2]. Despite the introduction of proton pump inhibitors, which has reduced the incidence of PPU, the surgical mortality of PPU remains high and is still a challenge for surgeons [3]. For evaluating the surgical risks and predict outcomes, the most common risk assessment tool for PPU is the Peptic Ulcer Perforation (PULP) score [4]. Although patient age and pre-existing comorbidities are evaluated in the scoring system, it does not assess the patient’s functional performance metrics, such as skeletal muscle strength. Sarcopenia is a progressive and generalized skeletal muscle disorder that is common in older individuals and is associated with multiple comorbidities [5–7]. It has also been well established as an independent risk factor for surgical outcomes [8–10]. However, it is apparently impractical to use traditional tools such as questionnaires, measurements of grip strength, dual-energy X-ray absorptiometry (DXA), and bioelectrical impedance analysis (BIA) to diagnose sarcopenia during abdominal emergencies [6]. Hence, abdominal computed tomography (CT), which is often used in the diagnosis of abdominal emergencies, has been introduced to obtain measurements of the skeletal muscle and psoas muscle [11, 12]. These measurements may be useful for the surgeon for preoperative assessment and planning [9, 13, 14].

As the association between sarcopenia and the outcome of PPU surgery is still unclear, the aim of this study was to evaluate the usefulness of skeletal muscle mass as measured on CT in assessing the surgical outcomes of older patients with PPU.

## Methods

### Study design and patient selection

This is a single institution, retrospective case–control study. A total of 347 older patients (≥ 65 years old) with PPU were admitted to Chang Gung Memorial Hospital between January 2011 and December 2016. The patients’ electronic medical records were reviewed for information on demographics, duration from the symptom onset to hospital admission, duration from the emergency room (ER) arrival to operation, the American Society of Anesthesiologists (ASA) score, the peptic ulcer perforation (PULP) score, comorbidities, preoperative laboratory tests, operative findings, operative time, blood loss, length of hospital stay, ICU stay, complications and mortality. In addition, the patients were further divided into PULP scores ≤7 or PULP scores>7 for subgroup analysis [4]. A serious postoperative complication was defined as Clavien–Dindo Classification > 3 in this study.

Patients who had a pathological diagnosis of malignant disease (*n*=9), had previously received surgery for PPU or any other gastric surgery (*n*=18), underwent conservative treatment without surgery (*n*=13), had incomplete medical records (*n*=29), and lacked a preoperative CT (*n* = 130) were excluded.

### CT image processing

The patients’ preoperative CT studies were collected in Digital Imaging and Communications in Medicine (DICOM) format. The CT images were anonymized and further processed using analytic morphomics as previously described [15, 16]. In this study, cross-sectional areas of skeletal muscle were measured by evaluating the slice at the level of the inferior aspect of the L3 vertebral body. The mean pixel intensity of the muscle pixels (defined as -29 to 150 HU) [7, 17] at the selected muscle was measured to obtain the mean muscle density. The muscle areas were normalized by the patient’s height and were adjusted by the mean muscle density to obtain a skeletal muscle gauge (SMG, cm^2^ HU/m^2^), which was first described by Weinberg et al. [18] Sarcopenia was defined as an L3 skeletal muscle gauge (L3 SMG) lower than 1490cm^2^ HU/m^2^ in males and 1085 cm^2^ HU/m^2^ in females, and these values were based on a previous study (eTable 1) [19].

$$\mathrm L3\;\mathrm{Skeletal}\;\mathrm{muscle}\;\mathrm{gauge}\;\left(\mathrm L3\;\mathrm{SMG}\right)\;=\frac{\mathrm{cross}-\mathrm{sectional}\;\mathrm{skeletal}\;\mathrm{muscle}\;\mathrm{area}\;\mathrm{at}\;\mathrm L3\;\mathrm{level}\;\left(\mathrm{cm}^2\right)\;\mathrm X\;\mathrm{mean}\;\mathrm{muscle}\;\mathrm{density}\;\left(\mathrm{HU}\right)}{\mathrm{subject}'\mathrm s\;\mathrm{height}^2\;\left(\mathrm m^2\right)}$$

### Follow-up

A surviving patient was defined as a patient who was discharged and followed up in outpatient clinic. Proton pump inhibitors were prescribed for 8-12 weeks. Surveillance endoscopies were performed after 8 to 12 weeks to rule out the other etiologies for the perforated ulcers. Triple therapy was prescribed once *H. pylori* infection was confirmed.

### Statistical analysis

SPSS 24.0 (SPSS Inc., Chicago, IL, USA) was used for statistical analysis. Descriptive statistics are presented as numbers and percentages for categorical variables and as the means, standard deviations, minima and maxima for numerical variables. For comparisons between the two groups, the independent T test was used for numerical variables, the Pearson chi-square test was used for large-sample-sized categorical variables, and Fisher’s exact test was used for small-sample-sized categorical variables. Kaplan–Meier life table analysis was used to calculate the patient-specific survival times. Differences in the survival, after being stratified by sarcopenia, were compared by the log-rank test. Univariate and multiple logistic regression analyses were performed taking “30-day mortality” as the outcome measure in the PULP > 7 group. A *p* value < 0.05 was considered statistically significant.

## Results

From January 2011 to December 2016, 146 patients were included in the study. The patient characteristics are listed in Table 1. The mean age was 77.9 ± 7.0 years old, and the mean BMI was 23.18 ± 4.16 kg/m^2^. The mean PULP score was 8.1 ± 2.1. A total of 128 (87.7%) of the enrolled participants underwent laparotomy. A total of 105 (71.9%) perforations were located in the stomach, and 41 (28.1%) were located in the duodenum. A total of 131 (89.7%) simple PPU closures and 15 (10.3%) gastrectomies were performed. Overall, the 30-day mortality rate was 10.3%, and the complication (Clavien–Dindo Classification > 3) rate was 19.9% in the cohort. The mean L3 SMG of the cohort was 1245.37 ± 481.42 cm^2^-HU/m^2^. Overall, 54.8% of patients were defined as sarcopenic by using the sex-specific L3 SMG cutoff values.

**Table 1:** Characteristics of the older patients with perforated peptic ulcers

**Characteristic (*n* = 146)**	***Mean ±SD; n(%)***
Age (years)^a^	77.9 ± 7.0
Sex *(n, %)*	
Male	75 (51.4%)
Female	71 (48.6%)
BMI (kg/m^2^)^a^	23.18 ± 4.16
PULP score^a^	8.1 ± 2.1
Morphomics Variables	
L3 SMG (cm^2^-HU/m^2^)^a^	1245.37 ± 481.42
Sarcopenia *(n, %)*	82 (54.8%)
Operative Variables	
Laparotomy/laparoscopy *(n, %)*	128 (87.7%/12.3%)
Operative method *(n, %)*	
Simple closure	131 (89.7%)
Gastrectomy	15 (10.3%)
Perforation site *(n, %)*	
Gastric	105 (71.9%)
Duodenum	41 (28.1%)
Outcome	
30-day Mortality *(n, %)*^a^	15 (10.3%)
Serious Complications *(n, %)*^b^	29 (19.9%)
Length of Stay (days)^a^	17.7 ± 12.4
Length of ICU Stay (days)^a^	5.1 ± 7.2

*BMI* body mass index, *PULP score* peptic ulcer perforation score, *L3 SMG* L3 skeletal muscle gauge, *ICU* intensive care unit

^a^*Mean ± SD*

^b^Serious complication was defined as Clavien–Dindo Classification > 3

Because the PULP score has been used to identify high-risk patients [4, 20, 21], we further divided the patients into the following two subgroups: patients with a PULP score ≤ 7 and those with a PULP score > 7 (Table 2). In the PULP score ≤ 7 group, sarcopenic patients (*n*=29) shared similar characteristics with nonsarcopenic patients (*n*=35), except the sarcopenic patients were older (79.3 ± 5.8 vs. 75.3 ± 6.4 years old, respectively, *p*=0.012). The length of hospital stay was significantly longer in the sarcopenic group (11.9 ± 8.5 days vs. 18.1 ± 15.4 days, *p*=0.041). Otherwise, there were no significant differences in the 30-day mortality or serious complication rates between sarcopenic and nonsarcopenic patients. However, in the PULP > 7 group, the sarcopenic patients (*n*= 51) were older (80.8 ± 7 years old vs. 74.7 ± 6.6 years old, p<0.001) and were more likely to have a previous peptic ulcer history (31.4% vs. 9.7%, *p*=0.024) than the nonsarcopenic patients (*n*=31). In addition, the sarcopenic group had a significantly higher 30-day mortality rate (25.5% vs. 3.2%, *p*=0.009) and serious complication rate (37.2% vs. 12.9%, *p*=0.017) than the nonsarcopenic group. Notably, there were no differences between the BMIs of the sarcopenic and nonsarcopenic patients in either of the groups.

**Table 2:** Demographics and outcomes of the subgroup analysis based on the PULP score and sarcopenia

**Variable**	**PULP score <= 7**			**PULP score > 7**		
	**Without sarcopenia (*n*=35)**	**Sarcopenia (*n*=29)**	***p value***	**Without sarcopenia (*n*=31)**	**Sarcopenia (*n*= 51)**	***p value***
Age (years)^a^	75.3 ± 6.4	79.3 ± 5.8	^#^0.012^*^	74.7 ± 6.6	80.8 ± 7	^#^0.000^*^
Male *(%)/*female *(%)*	19 (54.3)/16 (45.7)	10 (34.5)/19 (65.5)	^!^0.113	20 (64.5)/11(35.5)	26 (51.0)/25 (49.0)	^!^0.231
BMI (kg/m^2^)^a^	22.61 ± 4.41	23.06 ± 4.1	^#^0.676	23.7 ± 4.42	23.35 ± 3.92	^#^0.711
PULP score^a^	6.1 ± 1	6.4 ± 0.5	^#^0.145	9.3 ± 1.7	9.7 ± 1.6	^#^0.308
Peptic ulcer hx *(%)*	9 (25.7)	5 (17.2)	^!^0.414	3 (9.7%)	16 (31.4%)	^!^0.024^*^
Comorbidity *(%)*	23 (65.7)	21 (72.4)	^!^0.565	27 (87.1)	43 (84.3)	^!^1.000
Laparotomy/Laparoscopy *(%)*	24 (68.6)/11 (31.4)	25(86.2)/4 (13.8)	^!^0.097	29 (93.5)/2 (6.5)	50 (98.0)/1 (2.0)	^!^0.554
Gastrectomy/Simple closure *(%)*	2 (5.7)/33 (94.3)	3(10.3)/26 (89.7)	^†^0.651	4 (12.9)/27(87.1)	6 (11.8)/45 (88.2)	^†^1.000
Gastric/Duodenum *(%)*	24 (68.6)/11 (31.4)	24 (82.8)/5 (17.2)	^!^1.000	19 (61.3)/12 (38.7)	37 (74.0)/13 (26.0)	^!^0.587
Outcome						
30-day mortality *(%)*	1 (2.9)	0 (0.0)	^†^1.000	1 (3.2)	13 (25.5)	^!^0.009^*^
Length of stay (days)^a^	11.9 ± 8.5	18.1 ± 15.4	^#^0.041^*^	19.6 ± 11.3	21.8 ± 12.1	^#^0.481
Length of ICU stay (days)^a^	1.8 ± 1.8	6.0 ± 10.4	^#^0.062	6.7 ± 8.5	6.4 ± 5.0	^#^0.836
Serious Complication *(%)*^b^	4 (11.4)	2 (6.9)	^†^0.536	4 (12.9)	19 (37.3)	^!^0.017^*^
Leakage *(%)*	1 (2.9)	0 (0.0)	^†^1.000	5 (16.1)	10 (19.6)	^!^0.693
Cardiac complication *(%)*	2 (5.7)	3 (10.3)	^†^0.651	7 (22.6)	8 (15.7)	^!^0.434
Pulmonary complication *(%)*	4 (11.4)	6 (20.7)	^†^0.491	11 (35.5)	21 (41.2)	^!^0.608
Acute renal failure *(%)*	1 (2.9)	0 (0.0)	^†^1.000	3 (9.7)	8 (15.7)	^†^0.521

*BMI* body mass index, *PULP score* peptic ulcer perforation score, *L3 SMG* L3 skeletal muscle gauge, *ICU* intensive care unit

^a^*Mean ± SD*

^b^Serious complication was defined as Clavien–Dindo Classification > 3

^#^Student’s t test

^!^Pearson’s Chi-square test

^†^ Fisher’s exact test

^*^Statistically significant

**Fig. 1:**
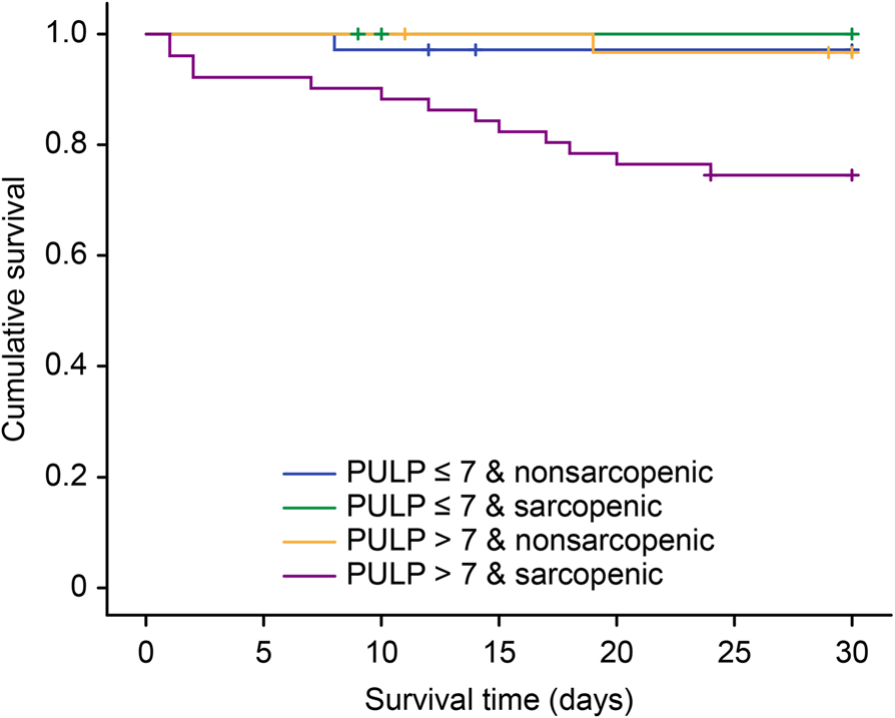
Kaplan–Meier estimated mortality curves for the subgroups stratified by sarcopenia and PULP score

To further analyze the impact of sarcopenia in the PULP > 7 group, a univariate analysis followed by a enter method multiple logistic regression analysis was performed (Table 3). Sarcopenia, defined by a low L3 SMG, was an independent risk factor for 30-day mortality in older PPU patients with a PULP score > 7 (OR: 11.05, CI: 1.03-118.7). The Kaplan–Meier survival curves showed that patients with both PULP score > 7 and sarcopenia had a significantly higher 30-day mortality rate than the other three groups (log-rank test, *p*<0.01) (Fig. 1).

**Table 3:** Logistic regression analysis of the 30-day mortality for patients with PULP score > 7

**Variable**	**Univariate**		**Multivariate**^†^	
	***p value***	***OR (95%CI)***	***p value***	***OR (95%CI)***
Age (years)	0.256	1.05 (0.97-1.13)	0.90	1.01 (0.91-1.12)
Male/female	0.099	2.73 (0.83-9.04)		
BMI	0.596	0.96 (0.83-1.11)		
PULP score	0.002*	1.81 (1.24-2.63)	0.02*	1.74 (1.09-2.81)
Comorbid active malignant disease or AIDS	0.103	2.88 (0.81-10.25)		
Comorbid liver cirrhosis	0.018*	6.40 (1.38-29.79)		
Concomitant use of steroids	0.999	0.00 (0.00-.)		
Shock on admission	0.968	0.97 (0.19-4.99)		
Time from perforation to admission > 24 hrs.	0.950	0.96 (0.27-3.42)		
Serum creatinine > 1.47 mg/dl	0.198	4.00 (0.49-32.98)		
ASA score 4 (compare to ASA3)	0.025*	6.00 (1.25-28.86)		
Peptic ulcer history	0.063	3.17 (0.94-10.74)		
Diabetes mellitus	0.226	2.08 (0.64-6.83)		
Hypertension	0.976	0.98 (0.30-3.26)		
Pulmonary disease	0.999	0.00 (0.00-.)		
Congestive heart failure	0.916	1.09 (0.21-5.71)		
Hospital to operation (Hr)	0.869	0.99 (0.93-1.06)		
Preoperative respiratory failure	0.006*	13.20 (2.13-81.72)	0.23	3.90 (0.43-35.53)
L3 SMG < -2.5 SD	0.029*	10.26 (1.27-82.94)	0.03*	11.05 (1.03-118.65)

*OR* odds ratio, *BMI* body mass index, *PULP score* peptic ulcer perforation score, *ASA* American Society of Anesthesiologists, *L3 SMG* L3 skeletal muscle gauge, *ICU* intensive care unit

^*^Statistically significant

^†^Multivariate logistic regression was performed using the entered method, which included variables that had a p-value less than 0.05 in univariate logistic regression and clinically important variables

Patients were categorized into four groups; PULP ≤ 7 without sarcopenia, PULP ≤ 7 with sarcopenia, PULP > 7 without sarcopenia and PULP > 7 with sarcopenia. PULP > 7 with sarcopenia group increase 30-day mortality compared to the other 3 groups

## Discussion

In the era of proton pump inhibitors and endoscopic treatment, the incidence of peptic ulcers has greatly decreased. However, the number of PPU cases has remained steady due to an increasing usage of nonsteroid anti-inflammatory drugs accompanied by an increasing patient age [1, 2]. Additionally, the mortality rate of PPU has remained high at approximately 10% [3, 22]. Traditionally, the PULP score has been widely used to predict the mortality rate of PPU. However, it only evaluates the patient’s preoperative risks, such as age, BMI, and preexisting comorbidities without assessing the physical performance of these patients, such as sarcopenia. Although sarcopenia is not an uncommon disorder in older individuals, to our knowledge, this is the first study to demonstrate the significance of sarcopenia on PPU surgery in this group.

Sarcopenia is defined as an age-related skeletal muscle disorder, characterized by decreasing muscle mass, quality, strength, and functional performance [6, 23]. According to the revised European Working Group on Sarcopenia in Older People (EWGSOP 2), sarcopenia is diagnosed by a low muscle strength and a low muscle quantity or quality and is considered severe when a patient has a low physical performance [6]. Aside from helping diagnosing PPU, some authors have also shown that preoperative CT images can also be used to assess sarcopenia. Recently, CT-measured skeletal muscle cross-sectional areas at the third lumbar (L3) vertebra have been introduced for the assessment of sarcopenia [16, 24, 25]. These CT-measured muscle indices are an effective tool to identify sarcopenia and are related to adverse outcomes in both cancerous (such as lung, gastrointestinal tract, and gynecological cancers) and noncancerous diseases (such as aortic valve implantation and liver disease) [26–28]. In the current study, the skeletal muscle mass was measured at the L3 level [28–30]. Instead of using the subject’s height alone to normalize the muscle area, the mean muscle density was also used for further standardization. This is in line with the revised guideline of EWGSOP2 regarding the concept of muscle gauges, which emphasizes the importance of both the muscle quality and muscle quantity. Several studies have demonstrated a stronger association between frailty and L3 skeletal muscle gauge, as opposed to the traditional muscle index [31, 32]. Furthermore, there have been proposals for a correlation between L3 muscle index, density, and extremities (thigh) muscle mass [33]. Muscle gauge has been identified as a significant risk factor for adverse outcomes. For instance, Lu et al. [34] discovered that a low total psoas gauge (TPG) was linked to unfavorable outcomes in gastric cancer patients who received radical gastrectomy. In our previous study, we found that a low muscle gauge had a negative predictive value for older patients with abdominal surgical emergencies [14, 19].

The PULP score was first described by Møller et al. [4], and is useful in predicting PPU mortality, demonstrating a sensitivity of 62.5% to 92.9% and a specificity of 58.3% to 87.3% [21]. However, only one-quarter to one-third of the mortalities could be predicted in the high-risk group (PULP > 7) [35]. Although an age older than 65 years is an integral part of the PULP score (3 points), our study suggested that age alone is not an independent risk factor for mortality. The physiological status of the older patients was an important factor as well, particularly if the patient had a PULP score >7. Our study showed that for older PPU patients with a PULP score > 7, sarcopenia was not only strongly associated with a higher rate of severe complications (37.2% vs. 12.9%, *p*=0.017) but also a strong independent risk factor for 30-day mortality (OR 11.62, CI 1.2-110.8, *p* = 0.03).

Interestingly, the surgical outcomes between the sarcopenic and nonsarcopenic patients did not differ in the low-risk (PULP ≤ 7) group. The results were consistent with our previous work that sarcopenia may have less impact in less severe forms of abdominal emergencies (such as appendicitis and cholecystitis) but have a much more significant impact in severe diseases (such as hollow organ perforation and mesenteric ischemia) [14].

Evaluating the adverse effects of sarcopenia in high-risk patients provides a better risk assessment and may alter treatment outcomes [36]. A systematic review showed strong evidence that exercise interventions improved the muscle strength and mass as well as postural balance in older adults with sarcopenia [37–39]. The 2018 International Clinical Practice Guidelines for sarcopenia recommend nutritional support, especially protein support, in sarcopenia patients [40]. Large-scale trials, such as the European SPRINTT trial (NCT02582138), are now underway to evaluate the effect of both physical activity and nutritional supplementation in sarcopenia patients [36, 41]. Further study will be needed to confirm the long-term outcomes in older sarcopenic patients with abdominal surgical emergencies.

Similar to our previous work, BMI was not a predictor of the postoperative outcomes of the older PPU patients [14]. This was not surprising because BMI only assesses the patient’s body size and cannot differentiate body compositions. On the other hand, the advancement of CT imaging and software helps to obtain accurate and precise measurements of body compositions. We believe that surgical planning and perioperative treatment for high-risk patients could be further optimized by measuring their CT-muscle gauge.

There are several limitations of this study. This was a retrospective single-center study. A prospective study is needed to confirm the utility of muscle gauges in PPU outcome prediction. The retrospective and emergent nature of our study also limited the ability to evaluate the correlation of SMG measured on CT and the other traditional function assessments of sarcopenia. Finally, external validation, such as with a multi-institutional study, might also be needed. However, this study still provided a promising prognostic value for the use of muscle gauges in older PPU patients.

## Conclusions

Aside from the traditional scoring systems, sarcopenia, defined as a low L3 SMG measured on CT, provides an extra value in predicting the outcome of high-risk older patients presenting with PPU.

## Supplementary Information

**Additional file1:** **eTable 1. **Cutoff Values of the Skeletal Muscle Gauge at the L3 Level in Asian Patients.

## Data Availability

The datasets analyzed in the current study are not publicly available due to issues related to institutional policy but are available from the corresponding author on reasonable request.

## Abbreviations

AIDS: Acquired immune deficiency syndrome
ASA: American Society of Anesthesiologists
AUROC: Area Under the Receiver Operating Characteristic curve
BIA: Bioelectrical Impedance Analysis
BMI: Body Mass Index
DXA: Dual-energy X-ray absorptiometry
EWGSOP: European Working Group on Sarcopenia in Older People
HU: Hounsfield unit
ICU: Intensive Care Unit
LOS: Length of stay
(MD)CT: (Multidetector) computed tomography
NSAID: Non-Steroidal Anti-Inflammatory Drug
L3 SMG: L3 Skeletal muscle gauge, in HU-cm2/m2
L3 SMI: L3 Skeletal muscle index, in HU-cm2/m2
PPU: Perforated peptic ulcer
PPI: Proton pump inhibitor
PriME: Perioperative management of elderly patients
PULP: Peptic ulcer perforation
SD: Standard deviation
TPG: Total psoas gauge
