# Supplementary material for: Sarcopenia provides extra value outside the PULP score for predicting mortality in older patients with perforated peptic ulcers
Source: BMC Geriatr. 2023 May 4;23:269. doi: 10.1186/s12877-023-03946-7 (PMC10161495; doi:10.1186/s12877-023-03946-7)
Supplement: Supplementary file 1 — Additional file1: eTable 1. Cutoff Values of the Skeletal Muscle Gauge at the L3 Level in Asian Patients. [file 12877_2023_3946_MOESM1_ESM.docx]

**eTable 1. Cutoff Values of the Skeletal Muscle Gauge at the L3 Level in Asian Patients**

| Morphomic Variables | Sex | *Mean ± SD* | *Mean - 2.5 SD* |
| --- | --- | --- | --- |
| L3 SMG  (cm^2^-HU/m^2^) | M | 2801.59±524.33 | 1490.77 |
|  | F | 2102.93±407.15 | 1085.05 |

L3 SMG: L3 Skeletal muscle gauge
